# Emergency physician attended calls that could have been covered by advanced emergency medical technicians in Austria: Results of the Austrian Emergency Day 2025 audit

**DOI:** 10.1007/s00508-026-02722-y

**Published:** 2026-03-05

**Authors:** Helmut Trimmel, Romana Erblich, Christian Anzur, Stefan Dressler-Stross, Wolfgang Voelckel, Mario Krammel, Armin Krösbacher, Berndt Schreiner, Christian Fohringer, Markus Gschanes, Gerhard Prause, Martin W. Dünser

**Affiliations:** 1 Department of Anaesthesia, Emergency and General Intensive Care Medicine, University Hospital Wiener Neustadt, Wiener Neustadt, Austria; 2 Karl Landsteiner Institute for Emergency Medicine, Wiener Neustadt, Austria; 3 Department of Anaesthesiology and Intensive Care Medicine, Kepler University Hospital and Johannes Kepler University, Krankenhausstrasse 9, 4020 Linz, Austria; 4 Institute for Mathematics and Statistics, Faculty of Engineering, Fachhochschule Wiener Neustadt, Wiener Neustadt, Austria; 5 Institute for Scientific Methods and Market Research, Faculty of Economics, Fachhochschule Wiener Neustadt, Wiener Neustadt, Austria; 6 Department of Anaesthesiology and Critical Care Medicine, AUVA Trauma Center Salzburg, Academic Teaching Hospital of the Paracelsus Private Medical University, Salzburg, Austria; 7 Emergency Medical Service Vienna, Vienna, Austria; 8 Department of Anaesthesiology and Critical Care Medicine, Innsbruck Medical University, Innsbruck, Austria; 9 Red Cross Lower Austria, Tulln, Austria; 10 Notruf Niederösterreich GmbH, St. Poelten, Austria; 11 Arbeitsgemeinschaft für Notfallmedizin, Graz, Austria

**Keywords:** Interventions, Competencies, Training, Emergency medical services, Inter-hospital transfers, Telemedicine

## Abstract

**Background:**

Emergency physician-staffed emergency medical services (EMS) in Austria frequently attend low priority calls, raising concerns about over-triage and future workforce sustainability.

**Objective:**

To quantify the proportion of emergency physician-attended calls that could have been managed by advanced emergency medical technicians (AEMT), with or without telemedical physician support, and evaluate current AEMT practice and training needs.

**Methods:**

This prospective, nationwide audit collected data from public physician-staffed EMS in Austria over a 24‑h period. Emergency physicians documented call characteristics, NACA (National Advisory Committee for Aeronautics) severity scores, AEMT presence, actual and potential EMT or AEMT interventions, accuracy of severity assessment and their judgement regarding the necessity of on-scene physician involvement.

**Results:**

Out of 149 physician-staffed EMS, 88 (59.1%) participated recording 313 calls and 273 emergency calls, 25 interhospital transfers and 15 telemedical emergency physician consultations were included. Emergency physicians assessed that 53.4% (95% confidence interval, CI 46.8–60.0%) of emergency calls and 48.0% (95% CI 28.4–67.6%) of interhospital transfers could have been managed by AEMTs. Approximately one third of these would have required telemedical physician support. The AEMTs arrived on the scene before emergency physicians in 75.1% of emergency calls and correctly assessed disease severity in 88.4% of cases and one or more additional interventions could have been set by AEMTs before emergency physician arrival in 48.0% of emergency calls. Most physicians supported expanding AEMT competencies and telemedical physician services.

**Conclusion:**

The results of this nationwide audit suggest that more than half of emergency calls and interhospital transfers attended by emergency physicians in Austria could have been managed by AEMTs with or without telemedical emergency physician support.

**Supplementary Information:**

The online version of this article (https://doi.org/10.1007/s00508-026-02722-y) contains supplementary material, which is available to authorized users.

## Introduction

In Austria, emergency medical services (EMS) operate as a two-tiered model consisting of ambulances staffed by predominantly volunteer emergency medical technicians (EMTs) or advanced EMTs (AEMTs) and emergency physician-staffed rapid response units [1–3]. Due to differences in federal legislature, the number and ratio of available EMTs/AEMTs as well as the dispatch pattern of ambulances (combined use for patient transport services and emergency medical services versus separation of patient transport services and emergency medical services) vary considerably between Austrian states [4]. Emergency physician-staffed units are dispatched to the scene in addition to EMTs or AEMTs in cases where a potentially or actually life-threatening event is suspected [1–3]. In 2024, the first telemedical emergency physician-staffed service operating 24/7 was established. This service currently supports EMTs or AEMTs in several Austrian states during low-acuity emergency calls or emergency calls unattended by emergency physician-staffed response units.

A recent nationwide audit revealed that the rate of trauma calls and severity of emergency cases attended by emergency physicians in Austria were low, median National Advisory Committee for Aeronautics (NACA) severity score of 3 (interquartile range, IQR 3–4) [5, 6]. The study further indicated that 53.9% of prehospital emergency calls, to which Austrian EMS physicians are dispatched, were rated as over-triage and might have been covered by nonphysician staff, such as better trained AEMTs or paramedics. In view of future demographic changes affecting both the general Austrian population and healthcare staff including emergency physicians [7, 8], it has been projected that the number of operating prehospital emergency physician-staffed units in Austria will decrease [9]. To prepare the Austrian EMS for these changes, it is important to better understand the current practice and interventions delivered by EMTs or AEMTs as well as the true proportion and characteristics of emergency physician-attended calls which could be covered by AEMTs in the future. This information should also help to define educational needs and inform the future training curricula of AEMTs in Austria.

In this study, we examined the proportion of emergency physician-attended calls that, in their opinion, could have also been handled by AEMTs. Additionally, we sought to evaluate interventions of EMTs or AEMTs during emergency physician-attended calls and collect opinions of emergency physicians on AEMT practice and training. Doing so, we aimed to quantify the potential proportion of emergency physician-attended calls that could be covered by AEMTs in the future and to identify practical as well as educational implications to improve EMS in Austria.

## Material and methods

The Austrian Emergency Day 2025 audit was a web-based, prospective, observational study, conducted under the auspices of the Austrian Society of Anaesthesiology, Resuscitation, and Intensive Care Medicine (ÖGARI). Data acquisition took place during a 24‑h period from 30 October 2025 at 07:00 a.m. until 31 October 2025 at 06:59 a.m. In line with a previous audit evaluated by the Ethics Committee of Lower Austria (GS3-EK-12/808-2024), formal approval or written informed consent was not required, as participation in the audit was voluntary, all data were anonymized and no patient-related information was collected. The manuscript was prepared according to the updated STROBE checklist for reporting cohort studies [10].

The Austrian Emergency Day 2025 audit was funded by the Austrian Society of Anaesthesiology, Resuscitation, and Intensive Care Medicine (ÖGARI). Members of the emergency medicine section of the ÖGARI designed and performed the study. Patients or the public were not involved in the design, conduct, reporting or dissemination plans of this research.

### Study population

All physician-staffed EMS dispatched by public emergency control centers in Austria were eligible for participation in this audit. National, state and the society’s registers were used to identify all public physician-staffed EMS in Austria. Physician-staffed EMS, which operated only during the summer or winter season and were not in service on the audit day, were excluded. The Austrian Emergency Day 2025 was advertised online on the society’s website, during conferences and through personal contacts of members of the ÖGARI emergency medicine section. In addition, 3 weeks and 1 week prior to the audit, electronic invitation letters were sent to the physicians in charge of 149 physician-staffed EMS. Each of these letters contained a link to a web-based form, which was accessible for online data entry from 28 October 2024 until 3 November 2025.

### Data collection and study instrument

All study-related data were entered using the aforementioned online form. This data entry form contained 46 questions, which were divided into 2 parts: part one was completed by the physician in charge of each physician-staffed EMS before the audit day. This part collected information about the physician-staffed EMS base, the non-physician-staffed EMS in the operational area as well as the emergency physician’s opinion on current AEMT practice and training. Part two of the data entry form was completed after each emergency call during the 24‑h observation period and collected the following data: type of call (emergency call, interhospital transfer, telemedical support by emergency physician), type of dispatch (immediate, crew request), category of emergency, the NACA severity score (a scoring system to determine the severity of medical emergencies ranging from 1–7, with 1 representing a minor condition with no need for medical intervention and 7 representing death) [11], presence and sequence of arrival of EMTs or AEMTs on the scene, type of actual and potential interventions delivered by EMTs or AEMTs before arrival of the emergency physician team (as determined by the emergency physician), adequacy of the assessment of the patient’s disease severity by EMTs or AEMTs (as determined by the emergency physician) and estimation of the emergency physician whether the emergency call could have been managed by AEMTs. If the emergency physician considered that this was the case, it was further assessed whether this would have required telemedical emergency physician support or not. If the emergency physician assessed that the presence of an emergency physician was required at the scene, the reasons for this assumption were collected.

The first draft of the survey instrument was generated by the authors. The questionnaire subsequently underwent pilot testing by five experts in the field of prehospital emergency medicine, who evaluated it regarding flow, salience, acceptability and administrative ease. Based on their feedback the questionnaire was modified. Finally, it was again reviewed and approved by all authors.

The total number of emergency calls conducted by all physician-staffed EMS in Austria during the 24‑h observation period was retrieved from all public emergency control centers after completion of the audit.

### Definitions

In line with the Austrian EMT Act (*Sanitätergesetz—SanG*, BGBl. I Nr. 30/2002) [12], EMTs (*Rettungssanitäter*) were defined as persons who underwent a 100‑h theoretical and 160‑h practical training program in basic emergency medicine. The AEMTs (*Notfallsanitäter mit allgemeinen Notfallkompetenzen*) were defined as EMTs, who took an additional 210 h of theoretical training, 80 h of practical training in a hospital and 280 h of practical training on an emergency physician-staffed rapid response car. We did not differentiate between volunteer EMTs or AEMTs and employed EMTs or AEMTs.

### Study objectives

The primary objective of this study was to report the proportion of emergency physician-attended calls, which emergency physicians assessed could have been managed by AEMTs. Secondary objectives were: (1) differences in the proportion of emergency physician-attended calls that could have been managed by AEMTs across NACA severity scores, (2) EMT or AEMT interventions before arrival of the emergency physician team, (3) EMS practice in the operational area of the participating emergency physician-staffed EMS and (4) opinions of emergency physicians in charge about AEMT practice and training. Furthermore, we analyzed the primary and first secondary study endpoints separately for physician-staffed ground emergency medical services (GEMS) and helicopter emergency medical services (HEMS).

### Data processing and statistical analysis

After closure of the online data entry, all data were transferred into an electronic spreadsheet. Following plausibility checks, statistical analyses were conducted using the SPSS software program (IBM SPSS Statistics 30.0.0.0; IBM, Armonk, NY, USA). No imputation methods were used in cases of missing data. Descriptive statistical methods were used to analyze and report primary and secondary study objectives. All data are presented as absolute values with percentages, if not given otherwise.

## Results

Out of 149 physician-staffed EMSs 88 (59.1%) participated in the audit. These included 65 physician-staffed GEMS, 22 HEMS and 1 emergency physician-staffed telemedical EMS. All nine Austrian states were represented in the audit (Electronic Supplementary Material Table S1).

During the 24‑h observation period, 313 calls were entered into the database. This corresponded to 67.7% (313/462) of all emergency calls conducted by physician-staffed EMS in Austria on the audit day, 273 (87.2%) were emergency calls, 25 (8.0%) physician-accompanied interhospital transfers and 15 (4.8%) were emergency calls covered by the emergency physician-staffed telemedical EMS (Table 1). Of the emergency calls 40 were cancelled before the emergency physician team reached the scene. Medical emergencies were the most frequent category of emergencies for all types of emergency physician-attended calls. The median (interquartile range) NACA severity score was 3 (3–4) for emergency calls, 4 (3–5) for interhospital transfers and 2 (2–3) for telemedical physician support calls. Whereas approximately two thirds of physician-attended emergency calls were cancellations or calls with NACA severity scores ranging between 1 and 3, only one third of interhospital transfers had a NACA severity score < 4 (Table 1). Of the emergency calls 17 could not be classified as either GEMS or HEMS calls.

**Table 1:** Characteristics of emergency calls, interhospital transfers and EMS calls with telemedical physician support

		Emergency calls			Interhospital transfers	Telemedical physician support
		All	GEMS	HEMS	All	All
	*n*	273*	207	49	25	15
**Call cancellation**	*n* (%)	40 (14.7)	35 (16.9)	5 (10.2)	0 (0)	0 (0)
**Crew request**	*n* (%)	65 (23.8)	51 (24.6)	8 (16.3)	0 (0)	15 (100)
**Category of emergency**	*n* (%)					
*Medical*		118 (43.2)	85 (41.1)	22 (44.9)	14 (56.0)	10 (66.7)
*Trauma*		43 (15.8)	28 (13.5)	12 (24.5)	3 (12.0)	1 (6.7)
*Neurological*		34 (12.5)	25 (12.1)	6 (12.2)	3 (12.0)	1 (6.7)
*Pediatric (incl. trauma)*		9 (3.3)	8 (3.9)	1 (2.0)	1 (4.0)	2 (13.3)
*Surgical*		6 (2.2)	4 (1.9)	2 (4.1)	0 (0)	1 (6.7)
*Other*		22 (8.1)	21 (10.1)	1 (2.0)	4 (16.0)	0 (0)
*Missing*		41 (15.0)	36 (17.4)	5 (10.2)	0 (0)	0 (0)
**NACA severity score**	*n* (%)					
*1 (minor condition)*		11 (4.0)	8 (3.9)	1 (2)	1 (4.0)	2 (13.3)
*2 (condition manageable with outpatient care)*		44 (16.1)	36 (17.4)	4 (8.2)	2 (8.0)	6 (40.0)
*3 (condition requiring inpatient care)*		80 (29.3)	63 (30.4)	14 (28.6)	5 (20.0)	6 (40.0)
*4 (potentially life-threatening condition)*		61 (22.3)	38 (18.4)	17 (34.7)	10 (40.0)	0 (0)
*5 (life-threatening condition)*		22 (8.1)	15 (7.2)	5 (10.2)	7 (28.0)	1 (6.7)
*6 (ROSC following CPR)*		1 (0.4)	0 (0)	1 (2)	0 (0)	0 (0)
*7 (death)*		13 (4.8)	11 (5.3)	2 (4.1)	0 (0)	0 (0)
*Missing*		1 (0.4)	1 (0.5)	0 (0)	0 (0)	0 (0)
*Call cancellations*		40 (14.7)	35 (16.9)	5 (10.2)	0 (0)	0 (0)
**NACA-based adequacy of dispatch**	*n* (%)					
*Cancellations* *+* *NACA 1–3*		175 (64.1)	142 (68.6)	24 (49.0)	8 (32.0)	Not applicable
*NACA 4–7*		97 (35.5)	64 (30.9)	25 (51.0)	17 (68.0)	
*Missing*		1 (0.4)	1 (0.5)	0 (0)	0 (0)	

*CPR* cardiopulmonary resuscitation, *NACA* National Advisory Committee for Aeronautics, *ROSC* return of spontaneous circulation, *GEMS* ground emergency medical services, *HEMS* helicopter emergency medical services

*17 calls could not be classified as GEMS or HEMS calls. Therefore, the sum of GEMS and HEMS calls does not equal 273

**Fig. 1:**
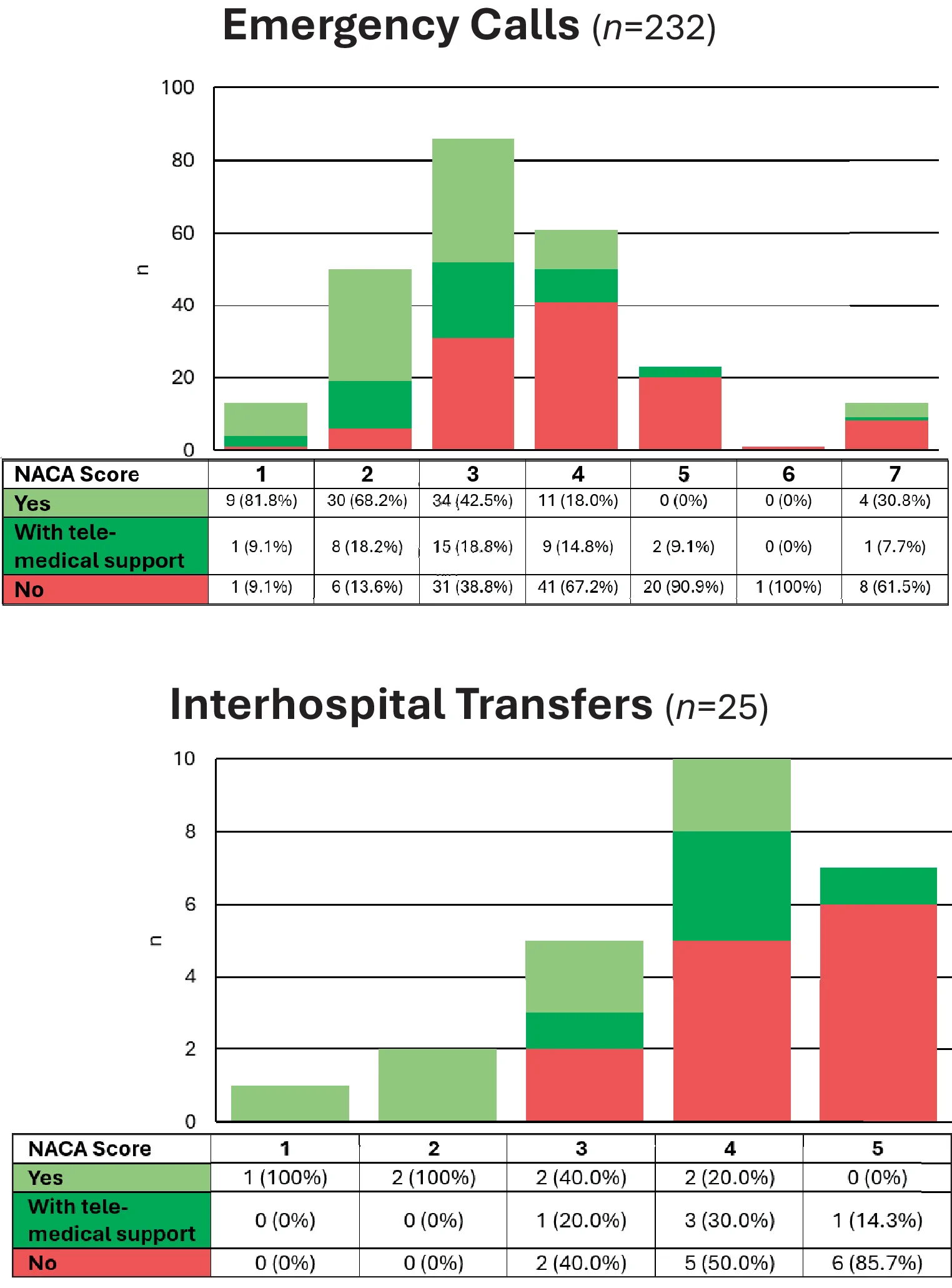
Stacked bar diagrams indicating the proportion of emergency physician-attended emergency calls (*upper*) and interhospital transfers (*lower*), which could have been managed by AEMTs with or without telemedical emergency physician support. *AEMT* advanced emergency medical technician

**Fig. 2:**
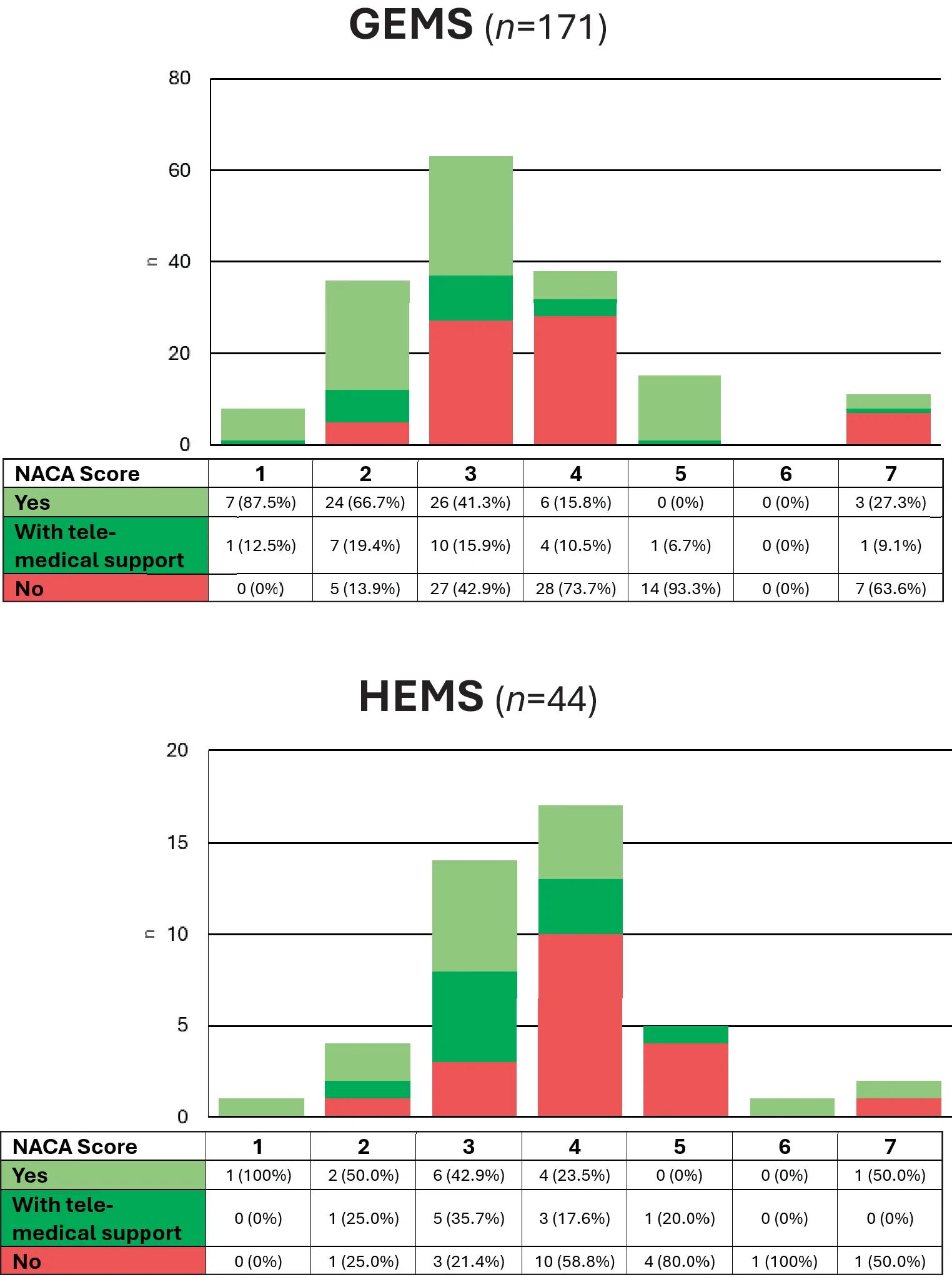
Stacked bar diagrams indicating the proportion of ground (*upper*) and helicopter (*lower*) emergency medical services-attended emergency calls, which could have been managed by AEMTs with or without telemedical emergency physician support. *GEMS* physician-staffed ground emergency medical services; *HEMS* helicopter emergency medical services

Out of 233 emergency physician-attended emergency calls with patient contact and 25 physician-accompanied interhospital transfers, assessments about whether calls or transfers could have been managed by AEMTs were available for 232 emergency calls (99.6%) and 25 interhospital transfers (100%). Emergency physicians assessed that 124/232 (53.4%; 95% CI 46.8–60.0%; GEMS: 52.6%; 95% CI 44.9–60.3%; HEMS: 54.6%; 95% CI 38.9–69.6%) of emergency calls and 12/25 (48.0%; 95% CI 28.4–67.6%) of interhospital transfers could have been managed by AEMTs. Based on the estimation of emergency physicians, 36 out of 124 of these emergency calls (29.0%; 95% CI 21.2–37.9%; GEMS: 26.7%; 95% CI 17.9–37.0%; HEMS: 41.7%; 95% CI 22.1–63.4%) and 5 out of 12 of these interhospital transfers (41.7%; 95% CI 13.8–69.6%) would have required telemedical emergency physician support. Out of 232 emergency physician-attended emergency calls 108 (46.6%; 95% CI 40.0–53.2%) and 13 of 25 emergency physician-accompanied interhospital transfers (52.0%; 95% CI 32.4–71.6%) were considered to be only manageable by an emergency physician on scene. The proportions of these estimations varied across NACA severity scores for emergency calls and interhospital transfers (Fig. 1). Although showing a similar pattern, these estimations varied between GEMS and HEMS (Fig. 2). Table 2 summarizes the reasons given by emergency physicians why they considered their presence was required in 108 emergency calls.

**Table 2:** Reasons why emergency physicians considered their on-scene presence required in 108 of 233 (46.4%) emergency calls with patient contact

	All emergency calls	NACA score I‑III	NACA score IV–VII
*n*	108	38	70
Tactical decisions*	45 (41.7)	15 (39.5)	30 (42.9)
*Analgesia*	36 (33.3)	16 (42.1)	20 (28.6)
*Basic airway procedures*^§^	20 (18.5)	3 (7.9)	17 (24.3)
*12-lead electrocardiogram*	19 (17.6)	5 (13.2)	14 (20.0)
*Medical history taking*	19 (17.6)	8 (21.1)	11 (15.7)
*Shock therapy*	14 (13.0)	0 (0)	14 (20.0)
*Endotracheal intubation*	12 (11.1)	0 (0)	12 (17.1)
*Sonography*	9 (8.3)	2 (5.3)	7 (10.0)
*Advanced cardiac life support*	7 (6.5)	0 (0)	7 (10.0)
*Pronouncement of death*	7 (6.5)	0 (0)	7 (10.0)
*Direct current cardioversion*	4 (3.7)	1 (2.6)	3 (4.3)
*Invasive blood pressure measurement*	4 (3.7)	0 (0)	4 (5.7)
*Noninvasive ventilation*	4 (3.7)	1 (2.6)	3 (4.3)
*Arterial blood gas analysis*	2 (1.9)	0 (0)	2 (2.9)
*Complex hemorrhage control*	2 (1.9)	1 (2.6)	1 (1.4)
*Palliative care*	2 (1.9)	0 (0)	2 (2.9)
*Chest decompression*	1 (0.9)	0 (0)	1 (1.4)
*Application of a pelvic splint*	1 (0.9)	0 (0)	1 (1.4)
*Other reasons*	24 (22.2)	12 (31.6)	12 (17.1)

Multiple responses were possible

*NACA* National Advisory Committee for Aeronautics

*including hospital priority admission

^§^excluding use of supraglottic airways and endotracheal intubation

An EMT or AEMT arrived on the scene in 75.1% of emergency calls before the emergency physician team. In 58.8% of emergency calls and 100% of telemedical physician support calls, an AEMT was present at the scene in addition to the emergency physician team. In 27.9% and 13.3% of emergency calls, no intervention was set by EMTs or AEMTs before arrival of the emergency physician team on scene or telemedical contact with the emergency physician, respectively. In 88.4% of emergency calls and 100% of telemedical physician support calls, EMTs or AEMTs correctly assessed the disease severity of the patient. In emergency calls with a NACA severity score < 4, EMTs or AEMTs underestimated the disease severity of the patient in 2.7% and overestimated it in 13.4% of cases. In emergency calls with a NACA severity score ≥ 4, the disease severity of the patient was underestimated in 2.0% and overestimated in 0% of cases. One or more interventions could have been delivered by EMTs or AEMTs before emergency physician team arrival in 48.0% of emergency calls and 20.0% of telemedical support calls (Table 3).

**Table 3:** Details on AEMT/EMT arrival, presence and interventions delivered on scene in emergency physician-attended calls

		Emergency calls with patient contact	Telemedical physician support
	*n*	233	15
**AEMT/EMT arrival on scene before emergency physician team**	*n* (%)	175 (75.1)	15 (100)
**Presence and arrival of AEMT on scene**	*n* (%)		
*Yes, before emergency physician team*		98 (42.1)	14 (93.3)
*Yes, arrival together with/after emergency physician team*		39 (16.7)	1 (6.7)
*No*		83 (35.6)	0 (0)
*Unknown*		13 (5.6)	0 (0)
**Calls with no interventions by AEMT/EMT before emergency physician team arrival**	*n* (%)	65 (27.9)	2 (13.3)
Interventions delivered by AEMT/EMT before emergency physician team arrival*	*n* (%)		
*Measurement of vital parameters*		135 (57.9)	12 (80.0)
*Oxygen delivery*		57 (24.5)	0 (0)
*Measurement of blood sugar*		55 (23.6)	7 (46.7)
*Positioning*		55 (23.6)	4 (26.7)
*Intravenous access*		40 (17.2)	9 (60)
*Basic life support*		8 (3.4)	0 (0)
*Bag valve mask ventilation*		8 (3.4)	0 (0)
*Protocol-guided drug therapy*		8 (3.4)	7 (46.7)
*Splinting (incl. cervical collar application)*		8 (3.4)	0 (0)
*Hemorrhage control*		6 (2.6)	0 (0)
*Analgesia*		4 (1.7)	2 (13.3)
*Laryngeal tube insertion*		1 (0.4)	0 (0)
**Assessment of the patient’s disease severity by AEMT/EMT**	*n* (%)		
*Correct*		206 (88.4)	15 (100)
*Disease severity overestimated*		20 (8.6)	0 (0)
*Disease severity underestimated*		6 (2.6)	0 (0)
*Missing*		1 (0.4)	0 (0)
**Calls with any intervention that could have been delivered by AEMT/EMT before emergency physician team arrival**	*n* (%)	131 (48.0)	3 (20.0)
Interventions that could have been delivered by AEMT/EMT before emergency physician team arrival*	*n* (%)		
*Intravenous access*		67 (28.8)	0 (0)
*Measurement of blood sugar*		55 (23.6)	0 (0)
*Measurement of vital parameters*		40 (17.2)	0 (0)
*Positioning*		31 (13.3)	1 (6.7)
*Protocol-guided drug therapy*		24 (10.3)	2 (13.3)
*Oxygen delivery*		18 (7.7)	0 (0)
*Analgesia*		15 (6.4)	3 (20.0)
*Splinting (incl. cervical collar application)*		13 (5.6)	1 (6.7)
*Laryngeal tube insertion*		5 (2.1)	0 (0)
*Hemorrhage control*		4 (1.7)	0 (0)
*Basic life support*		2 (0.9)	0 (0)
*Defibrillation*		2 (0.9)	0 (0)
*Bag valve mask ventilation*		2 (0.9)	0 (0)
*Endotracheal intubation without drugs*		1 (0.4)	0 (0)

*AEMT* advanced emergency medical technician, *EMT* emergency medical technician

*multiple responses possible

The physicians in charge of the 88 physician-staffed EMS reported details on EMS organization in their operational area (Table 4). Of the respondents 80% considered that an extension of AEMT competencies was warranted. The most frequent suggestion to improve AEMT training was more practical training in hospitals. An expansion of emergency physician telemedical support of AEMTs was supported by 71.6% of respondents.

**Table 4:** EMS details and emergency physicians’ opinions on AEMT practice and training

	*n*	88
**Details on EMS organization in operational area**		
Separation of patient transport service and EMS	*n (%)*	54 (61.4)
Emergency ambulances staffed with at least 1 AEMT	*n (%)*	25 (28.4)
Protocolized drug use by AEMT	*n (%)*	85 (96.6)
Involvement of EMS physicians in AEMT training	*n (%)*	51 (58.0)
**Emergency physician opinion on AEMT practice and training**		
Practice of protocolized drug uses by AEMT	*n (%)*	
*Too liberal*		7 (8.0)
*Appropriate*		54 (61.4)
*Too restrictive*		27 (30.7)
Expansion of AEMT competencies	*n (%)*	
*Extension warranted*		9 (10.2)
*Extension warranted pending improved AEMT training*		62 (70.5)
*Extension not warranted*		17 (19.3)
Suggestions to improve AEMT training	*n (%)*	
*More theoretical training*		52 (59.1)
*More practical training on EMS cars*		49 (55.7)
*More practical training on physician-staffed rapid response cars*		50 (56.8)
*More practical training in hospitals*		64 (72.7)
Support that a 3-year paramedic training is needed	*n (%)*	78 (88.6)
NACA severity score up to which adequately trained AEMTs should cover emergency calls without emergency physicians		
*2 (condition manageable with outpatient care)*		2 (2.3)
*3 (condition requiring inpatient care)*		44 (50.0)
*4 (potentially life-threatening condition)*		19 (21.6)
*NACA severity score not useful to make this decision*		23 (26.1)
Expansion of emergency physician telemedical support of AEMT/EMTs warranted	*n (%)*	63 (71.6)

*AEMT* advanced emergency medical technicians, *EMS* emergency medical service, *NACA* National Advisory Committee for Aeronautics

## Discussion

This nationwide audit included 313 emergency physician-attended calls on a single day in Austria. Both the cancellation rate as well as the ratio between emergency calls and interhospital transfers were comparable to the results of the Austrian Emergency Day 2024 audit [5]. Similarly, the categories of emergencies and the distribution of NACA severity scores compared well with results from the previous year. Although study data were only collected during a 24‑h period, they appear to consistently reflect the profile of calls attended by emergency physicians in Austria.

In the present audit emergency physicians judged that over half of all emergency physician-attended calls could have been managed by AEMTs with or without telemedical emergency physician support. This finding appeared more pronounced in physician-staffed GEMS than HEMS. In addition to an overall 14.7% cancellation rate, this proportion represents a substantial amount of emergency calls attended by emergency physicians and highlights the low acuteness of the majority of these calls. As we collected the subjective assessments of emergency physicians, this result can be objectivized by the finding that 64.1% of emergency calls in this audit were graded with a NACA severity score < 4, a finding in line with those of prior studies from Austria [5, 13]. Notably, the proportion of calls considered manageable by AEMTs was similar for interhospital transfers and emergency calls despite a higher median NACA severity score in interhospital transfers.

When evaluating the reasons why emergency physicians considered their presence on scene was required in 46.6% of emergency calls, it appears that better trained AEMTs might cover an even higher proportion of emergency calls than suggested by this audit. Accordingly, tactical decision making, analgesia, basic airway management and 12-lead ECG recording (e.g., with telemedical emergency physician interpretation) might represent topics which should be included into future AEMT curricula. Furthermore, organizational adjustments allowing priority hospital admissions (e.g., direct admissions to the catheter laboratory) also for AEMTs could obviate the need for emergency physician dispatches to the scene in further cases.

Importantly, > 90% of emergency physicians in charge considered the current practice of protocolized drug use by AEMTs in Austria appropriate or too restrictive. Accordingly, > 80% of respondents were in favor of expanding AEMT competencies. Notably, 88.6% of emergency physicians in charge supported a 3-year paramedic training program comparable to other European countries [14]. These results are a strong signal that Austrian emergency physicians advocate an improved AEMT training and expansion of competencies. The most frequent proposal to improve AEMT training was an extended practical training in hospitals.

Despite the fact that only one quarter of emergency physicians in charge reported that emergency ambulances in their operational area were staffed with at least one AEMT, the finding that AEMTs were present on scene in two thirds of emergency physician-attended calls indicates that emergency ambulances staffed with AEMTs are selectively dispatched; however, this result also emphasizes that further efforts are needed to reach the goal that all emergency ambulances in Austria are staffed by at least one AEMT. This is relevant, as a recent evaluation found that only 20% of all Austrian EMTs (*n* = 48,000; 75% volunteers) were trained as AEMTs [15].

In the majority of emergency calls, EMTs or AEMTs arrived on scene before the emergency physician team. Although interventions were set in 72.1% of calls, the fact that further interventions could have been delivered by EMTs or AEMTs in half of all calls highlights that improvements of both EMT and AEMT training are necessary. In this context, it is notable that EMTs or AEMTs correctly assessed the severity of the patient’s condition in most emergency calls, particularly those with a higher acuity. Underestimation of disease severity in the latter cases might have particularly devastating consequences.

Although telemedical emergency physician support accounted for only a small number of emergency calls, its role is relevant when discussing future task allocation in Austrian EMS. This service is currently used primarily for low-acuity emergencies. The finding that emergency physicians stated that 15.5% of all emergency calls handled by emergency doctors and 20.0% of transfers between hospitals accompanied by emergency doctors could have been handled by AEMTs with telemedical support underscores the need to also include emergency calls and interhospital transfers involving potentially or actually life-threatening emergencies into the spectrum of telemedical emergency physician services. This is reinforced by the broad support of emergency physicians for the expansion of telemedical emergency physician services.

The key strengths of this audit are its nationwide coverage and the high number of emergency calls recorded. On the other hand, it is a limitation that the number of interhospital transfers (*n* = 25) and telemedical physician support calls (*n* = 15) were low resulting in wide 95% confidence intervals. Second and as mentioned above, the observation period was limited to 24 h. Although we could reproduce the profiles of emergency calls from a similar audit conducted in 2024 [5], the short observation period still limits extrapolation of our results to other seasons of the year. Similarly, we are unaware how our results relate to the annual call profile of emergency physician-attended calls in Austria. Third, the primary outcome variable was based on the subjective assessment of emergency physicians and may have been influenced by individual opinions, experience and situational judgement of emergency physicians. Although the NACA severity score allowed some objectivation of these estimates, the NACA severity score itself might be prone to similar biases and has been criticized as an inappropriate tool to justify or refute the need for the presence of an emergency physician on scene. Fourth, we did not evaluate whether smartphone-activated community first responders, who can provide life-saving interventions prior to EMS arrival [16], were present on scene in cases of out-of-hospital cardiac arrests. Finally, it is important to remember that we only evaluated emergency physician-attended calls in this audit. We cannot determine the profiles and management of emergency calls without emergency physician involvement.

In conclusion, the results of this nationwide audit suggest that more than half of emergency calls and interhospital transfers attended by emergency physicians in Austria could have been managed by AEMTs with or without telemedical emergency physician support. Our findings underscore the need to improve AEMT training, expand telemedical emergency physician services and help to inform future AEMT educational curricula in Austria.

## Supplementary Information

ESM1: Supplementary material 1
