# Supplementary material for: Emergency physician attended calls that could have been covered by advanced emergency medical technicians in Austria: Results of the Austrian Emergency Day 2025 audit
Source: Wien Klin Wochenschr. 2026 Mar 5;138(17-18):558–67. doi: 10.1007/s00508-026-02722-y (PMC13585889; doi:10.1007/s00508-026-02722-y)
Supplement: Supplementary file 1 [file 508_2026_2722_MOESM1_ESM.docx]

**Electronic Supplementary Table S1.** Number and type of physician-staffed emergency medical services categorized by the nine federal states of Austria.

|  | **All** | **GEMS** | **HEMS** |
| --- | --- | --- | --- |
| **Austria** | **88 (100)*** | **65 (100)** | **22 (100)** |
| Burgenland | 3 (3.4) | 1 (1.5) | 2 (9.1) |
| Carinthia | 6 (6.8) | 4 (6.2) | 2 (9.1) |
| Lower Austria | 29 (33.0)* | 25 (38.5) | 3 (13.6) |
| Salzburg | 6 (6.8) | 2 (3.1) | 4 (18.2) |
| Styria | 9 (10.2) | 7 (10.8) | 2 (9.1) |
| Tyrol | 10 (11.4) | 6 (9.2) | 4 (18.2) |
| Upper Austria | 14 (15.9) | 11 (16.9) | 3 (13.6) |
| Vienna | 6 (6.8) | 5 (7.7) | 1 (4.5) |
| Vorarlberg | 5 (5.7) | 4 (6.2) | 1 (4.5) |

*, included 65 physician-staffed rapid response car emergency medical services, 22 helicopter emergency medical services and 1 telemedical emergency physician support service (in Lower Austria).

GEMS, physician-staffed ground emergency medical service; HEMS, helicopter emergency medical service.

Data are given as absolute numbers with percentages in parentheses.
